# Draft genome sequence of *Vreelandella neptunia* strain 04GJ23 isolated from the underwater Hawaii seamounts

**DOI:** 10.1128/mra.00882-24

**Published:** 2025-05-22

**Authors:** Brynne Darden, Gabriel Johnson, Grace Busch, Indu Sharma

**Affiliations:** 1 Hampton University 3726, Hampton, Virginia, USA; University of Southern California, Los Angeles, California, USA

**Keywords:** environmental microbiology, microbial ecology, microbial genomics

## Abstract

We report a draft genome sequence for *Vreelandella neptunia* strain 04GJ23 isolated from the underwater Hawaii seamounts. The whole-genome sequence will help understand the ecology and evolution of various ecotypes that are physiologically distinct from the surrounding environments.

## ANNOUNCEMENT

The *Vreelandella* genus, formerly classified as *Halomonas*, belongs to the family Halomonadaceae (1). This group of halophilic bacteria is ubiquitous and found in diverse environments, ranging from terrestrial to extreme marine environments like brine pools (2). *Vreelandella* bacteria have been shown to detoxify environmental pollutants and carcinogens and to degrade organic compounds and heavy metals in saline environments (2). Certain strains of *V. neptunia* are known to produce polyhydroxyalkanoates (PHA) used to synthesize bioplastics (3–5).

The deep-sea water sample NA138-059 was collected as part of expedition Luʻuaeaahikiikekumu - Ancient Seamounts of Liliʻuokalani Ridge (global positioning satellite coordinates latitude 31.612004, longitude −174.063672) at depth 2,246.41 m. The microbial enrichment was set up with potassium iodate (10 mM) in artificial seawater (6) media supplemented with yeast extract 0.04%, tryptone 0.2%, glycerol 0.3%, and MOPS 5 mM (SWC-KIO_3_). The enriched cultures were serially diluted and plated on SWC-KIO_3_ with 1.5% agar. A colony was streaked to obtain pure culture, the 16S gene was PCR amplified using 27F and 1492R primers, and sequenced using Sanger sequencing (7–10). The partial 16S gene sequence was compared using blastn and was 99.52% similar to *V. neptunia* accession number CP140255.1 (11). For whole genome sequencing, 1 mL of culture was grown in SWC-KIO_3_ for 1 day at 30°C and 100 rpm. The genomic DNA was isolated using the Quick-DNA Miniprep kit (Zymo Research, Orange, CA, USA). Invitrogen Qubit 4 Fluorometer and 1× dsDNA High-Sensitivity Assay kit (Thermo Fisher Scientific, Waltham, MA, USA) were used to quantify the gDNA. Genomic libraries were prepared using DNA extracts and the Nextera XT DNA Library Preparation kit (Illumina, San Diego, CA, USA) according to the manufacturer’s protocol. Libraries were quality checked using an Agilent 2100 Bioanalyzer and DNA High-Sensitivity kit and then pooled in an equimolar ratio. The pool was gel purified using a 2% agarose gel and the Qiagen QIAquick gel extraction kit (Qiagen, Germantown, MD, USA). Following purification, the pool was sequenced on an Illumina NextSeq 550 instrument using a Mid-Output v2.5 chemistry 300-cycle kit to produce 2× 150 bp reads.

**Fig 1:**
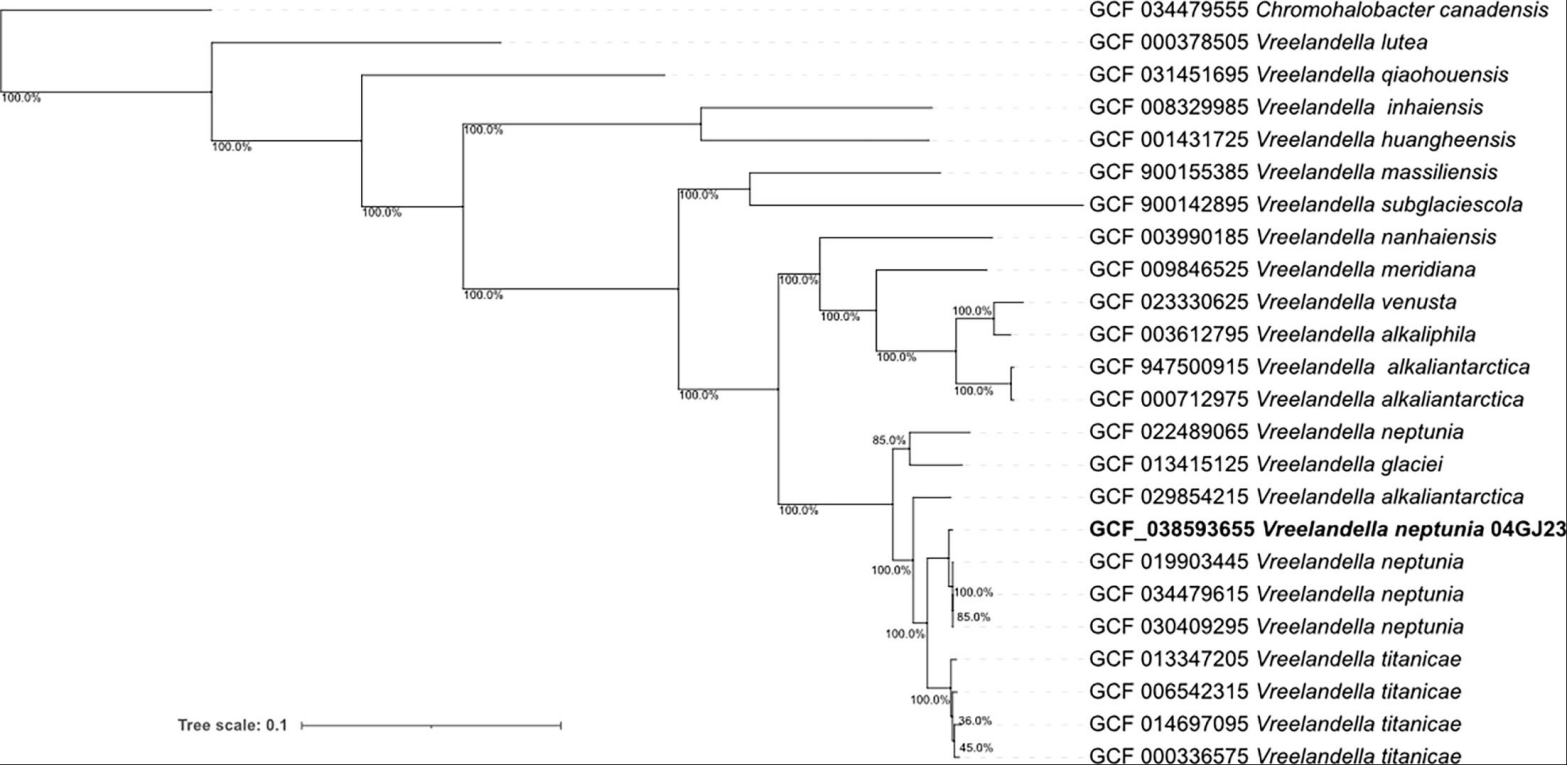
Phylogenomic tree of *V. neptunia* 04gj23 and related species, with *Chromohalobacter canadensis* as an outlier, was generated using 172 single-copy core genes for Gammaproteobacteria. Bootstrap values greater than 50% are indicated on branches based on 1,000 replicates. The scale bar represents the number of nucleotide changes per site.

For whole genome assembly, default parameters were used for all software unless otherwise specified. The raw data were quality checked with FASTQC v0.11.8 and has 10,609,478 × 2 reads (12). Trimmomatic v0.39 was used to trim the low-quality reads using the following parameters: LEADING:3 TRAILING:3 SLIDING-WINDOW:4:20 MINLEN:60, and a total of 5,036,390 reads were retained (13). The genome was assembled using SPAdes v3.13.0, achieving a final genome coverage of 329× (14). The assembly summary statistics were generated with QUAST v5.0.2 (15). The draft genome’s total sequence length is 4,827,065 bp, with 55 contigs (*N*50, 262,096  bp; *L*50, 8; *N*75, 123,549  bp; *L*75, with an average of 55.12% GC content. The genome was annotated using the NCBI Prokaryotic Genome Annotation Pipeline (16), which displayed 4,531 genes, 4,434 protein-coding genes, and 68 RNA genes. The average nucleotide identity-BLAST (ANIb) between the *Vreelandella* sp. 04GJ23 and *V. neptunia* (Accession number CP140255.1) is 98.11%, with 87.7% of the two genomes aligning (17). A phylogenetic tree (Fig. 1) was created using GToTree v1.7.00 (18–23), using 172 single-copy core genes for Gammaproteobacteria, with FastTree 2 v2.1.11 (24) used for inference. The phylogenomic tree presents genomic relationships of *V. neptunia* 04GJ23 and the family Halomonadaceae.

## Data Availability

This whole-genome shotgun project was deposited in NCBI’s GenBank under accession number JBCFYR000000000.1. Raw sequencing reads were deposited in NCBI’s Sequence Read Archive under accession number SRX25289493.
